# What should community organisations consider when deciding to partner with researchers? A critical reflection on the Zilla Budakattu Girijana Abhivrudhhi Sangha experience in Karnataka, India

**DOI:** 10.1186/s12961-020-00617-6

**Published:** 2020-09-11

**Authors:** Bridget Pratt, Tanya Seshadri, Prashanth N. Srinivas

**Affiliations:** 1 Centre for Health Equity, School of Population and Global Health, University of Melbourne, 207 Bouverie St., Carlton, Victoria 3053 Australia; 2 Institute of Public Health, 3009, II-A Main, 17th Cross, Krishna Rajendra Rd, Banashankari Stage II, Bangalore, Karnataka 560070 India; 3 Vivekananda Girijana Kalyana Kendra, BR hills, Chamarajanagar district, Karnataka, 571441 India

**Keywords:** community organisation, ethics, power, engagement, partnership, collaboration, health research, health systems research

## Abstract

**Background:**

Community organisations and community members are increasingly being involved in health research projects worldwide as part of the engagement movement. Achieving deeper forms of community engagement like partnership demands that decision-making power be shared with community partners. However, how can community partners assess if meaningful engagement and shared decision-making will be possible when approached by prospective research partners? In this paper, we explore how community organisations decide to join health research projects when approached by health researchers.

**Methods:**

Case study research was undertaken on a health systems research project in Karnataka, India called Participation for Local Action, which was carried out by local researchers in partnership with the Zilla Budakattu Girijana Abhivrudhhi Sangha, a community development organisation. In-depth interviews were conducted with the researchers, Sangha leaders and field investigators from their community.

**Results:**

Thematic analysis identified two main themes – ‘context’ and ‘deciding to engage’. The Sangha’s experience offers lessons to other community organisations that can help them when deciding to engage with researchers in terms of what features to look for in research partners and in proposed research projects, what requests to make of prospective research partners, and what sorts of outcomes or partnership agreements to accept. These lessons may be especially applicable in contexts where relationships of trust already exist between partners and where they have the skills to lead data collection and analysis.

**Conclusions:**

We hope that this guidance will help empower community organisations to select good research partners and promote more equitable partnerships between community partners and academic researchers.

## Background

Community engagement has long been promoted as a core element of participatory health research and there is rising consensus that community engagement is ethically and scientifically essential for all health research [1]. As such, research institutions, international research ethics guidelines and funding bodies now promote, or even mandate, community engagement as an important component of even ‘traditional’ non-participatory health research [2–4]. Funders of health systems research in low- and middle-income countries, for example, frequently require or expect engagement to be undertaken [5]. Community organisations (COs) and individual community members are thus increasingly being involved in health research projects worldwide.

Sharp and Foster [6] describe a spectrum of power sharing, from community dialogue through community consultation and approval to full partnership, where the latter implies shared decision-making throughout projects and the greatest community empowerment. Woolf et al. [7] contend that authentic engagement means involving those engaged as full partners and decision-makers when setting research project topics and questions, shaping project design, conducting data collection and analysis, and disseminating findings. Engaging communities who are considered disadvantaged or marginalised by social institutions and norms as collaborators or partners provides a path for making their voices and concerns more audible and visible in agenda-setting and in the production of scientific knowledge. It can help address epistemic injustice and generate research topics and questions that are more explicitly focused on improving access and affordability of healthcare and services for them [8–10]. Health systems research, for example, is “*driven by understanding of local contexts. At all stages of the research endeavour, from prioritization of research questions, to conceptualization and conduct of the research, to interpretation of and communication of findings HPSR will benefit from being embedded within a particular context and close engagement with local actors*” [11].

Yet, it is uncommon for COs and individual community members to be included in decision-making throughout health research projects in practice. Community partners, especially those from communities considered disadvantaged or marginalised, rarely initiate new research projects or are invited by researchers to have a say in setting agendas or designing research projects [12]. Indeed, even when invited to enter decision-making spaces, unequal power dynamics between COs/community members and researchers can give rise to tokenism – presence without voice and voice without influence, particularly for the most marginalised. Existing evidence confirms that, being female, being poor, having little education, living with a disability and/or belonging to certain ethnic groups mean that community members are listened to less or not at all in health priority-setting [13].

So how can COs and community members determine if shared decision-making will be possible when approached by prospective research partners? What are the key considerations for COs and community members when deciding whether to collaborate with researchers on a health research project? It is an important question to investigate because COs and community members need guidance on these matters just as much as researchers. Such guidance is essential to help empower them to select engagement opportunities that comprise full partnerships and that are likely to benefit the community they represent.

In this paper, we explore how COs decide to join health research projects when approached by health researchers and we do so in the context of a particular collaborative health systems research project where shared decision-making was achieved. Case study research was undertaken on the Participation for Local Action (PLA) project in Karnataka, India, to investigate how decision-making power was shared in priority-setting for the PLA project. In this paper, we report the case study data and then critically reflect on the key lessons it offers for other COs about how to assess whether meaningful engagement and shared decision-making is likely to occur with prospective research partners.

## Methods

### The case under study

The case study was performed as part of a larger ethics research programme that examines sharing power with marginalised communities in health research priority-setting. Cases of priority-setting processes were selected where marginalised groups were meaningfully engaged in choosing research project topics and questions and in designing interventions. The PLA project was one such case. It aimed to improve the implementation of safe motherhood services for the indigenous population in Chamarajanagar district in Karnataka state in southern India.

The PLA project ran from January 2015 to September 2016. Its research team consisted of seven members: a principal investigator from Vivekananda Girijana Kalyana Kendra (VGKK), an Indian non-governmental organisation (NGO); one co-investigator from the Institute of Public Health (IPH), an Indian research institution; one co-investigator from the Zilla Budakattu Girijana Abhivrudhhi Sangha (district Sangha); two co-investigators from other Indian research institutions; one co-investigator from a Belgian university; and two co-investigators from the Government of Karnataka. The Sangha is a district-level community development organisation representing the Soliga people living in Chamarajanagar district. The Soliga are an Indigenous population in India who have lived in the BR Hills region and surrounding forested regions of Chamarajanagar district in Southern Karnataka for centuries. The principal investigator, the IPH co-investigator and the district Sangha co-investigator were by far the most active research team members. The PLA project was undertaken in partnership with the district Sangha, which is comprised of 21 members [district Sangha leaders]. Chamarajanagar district is divided into four sub-districts (which are called taluks): Chamarajanagar, Gundlupet, Kollegal and Yelandur. Sangha organisations also exist at sub-district and village levels.

Several introductory meetings were conducted in 2015 between the VGKK and IPH researchers and district Sangha leaders. Once a decision to participate was made, a participatory action research process was designed and undertaken. The district Sangha recruited 10 field investigators from the Soliga community and a field supervisor to visit the district’s 148 Soliga villages and collect relevant data about the problems that households faced in accessing maternal and child health services. Data was jointly analysed by 3 district Sangha leaders and the principal investigator and 6 core themes were identified. In June 2016, a deliberative workshop was held and attended by 60 Sangha leaders (from district, sub-district and village levels) to reflect on the 6 themes, to prioritise amongst them and to develop community-led solutions to address them.

### Case study methods

Data were collected using a triangulation approach consisting of in-depth interviews (December 2018 and April 2019), document analysis and direct observation during a meeting between the researchers and district Sangha leaders (April 2019). Sampling was purposive; it sought to capture the perspectives of all types of stakeholders involved in the PLA project – researchers, Sangha leaders and field investigators from the Soliga community. Specific Sangha leaders and field investigators were suggested for interview by the district Sangha leader who supervised the field investigators. Efforts were made to select Sangha leaders and field investigators across genders and all four sub-districts as well as field investigators with and without Sangha affiliation, but this proved difficult.

A total of 14 semi-structured in-depth interviews were conducted with researchers (4 interviews), Sangha leaders from district and sub-district levels who participated in the introductory meetings and/or the deliberative workshop (5 interviews), and field investigators (5 interviews). Most field investigators who were interviewed were affiliated with the Sangha as either leaders or volunteers. In total, 4 women and 11 men were interviewed. To some extent, this reflected the fact that there were fewer female co-investigators and far fewer female Sangha leaders than male ones. Only 3 female Sangha leaders attended the initial meetings. Of the 10 Sangha leader and field investigator interviewees, 6 were from Yelandur sub-district, 3 from Kollegal sub-district and 1 from Chamarajanagar sub-district; 7 were Sangha leaders and 1 volunteered with the Sangha. Two interviewees were not part of the Sangha organisation.

Written informed consent was obtained from all participants. Following the technique of thick description, interview questions were open-ended [14] and a subset of the questions attempted to draw out interviewees’ experience with and perspectives on the introductory PLA project meetings. Interviewees were asked the following questions:

- What did the researchers tell you [the Sangha leaders] about the PLA project at your first meeting with them?
- What did you [the Sangha leaders] say at the meeting about the research topic of maternal and child health?
- How did the Sangha’s goals affect and shape the PLA project’s agenda?
- Was it a fair discussion or negotiation? How?

The question guide was translated to Kannada and then back-translated to English to ensure questions retained their original meaning.

All interviews were conducted in person at the IPH field station in the BR Hills in Chamarajanagar district, 13 by BP and 1 by NSN. Interviews with researchers were performed in English. Interviews with Sangha leaders and field investigators were performed in Kannada with the assistance of a research assistant (NSN). The interviews lasted between 63 and 126 minutes.

A meeting between the district Sangha leaders and researchers was observed by BP and NSN in April 2019 to supplement the interview data. This meeting was about the Towards Health Equity & Trasnsformative Action on tribal health (THETA) project, a subsequent project being conducted by the VGKK and IPH researchers and district Sangha. During the meeting, BP and NSN each took shorthand notes regarding who led the meeting, where people physically sat, who spoke, who was silent or listening, what questions were asked by Sangha leaders to the researchers, whether Sangha leaders challenged or disagreed with things the researchers said, who made decisions, whose voices were reflected in the decisions that were made, and whether consensus was reached. After the meeting, the two observers debriefed about what they had seen. As NSN spoke Kannada and BP did not, NSN’s observations largely held sway where there was a discrepancy. Project-related documents were also collected, including the PLA project final report; videos about the Soliga people, their history and culture; and a film made about the PLA project.

Interviews were transcribed verbatim and, where necessary, translated from Kannada to English. Thematic analysis of interview transcripts, notes from direct observation and debrief, the PLA final report, and notes from videos were undertaken by two coders in the following five phases: initial coding framework creation, coding, inter-coder reliability and agreement assessment, coding framework modification, and final coding of the entire dataset [15, 16].

### Authors’ positionality

The paper’s first author is an ethics researcher from a high-income country. She was not a member of the PLA project team. This case study was her first introduction to the Sangha and its processes as well as to the Soliga people, their history and their way of life. She does not speak Kannada.

The remaining two authors were part of the PLA project research team. Both are medical doctors and have worked in primary care at VGKK hospital. In 2009, they set up a long-term tribal health research field station embedded within the Soliga community and with linkages to local health departments, NGOs and indigenous COs. They have purposefully limited their participation in designing and conducting the case study. They provided input on the study’s recruitment scripts, Plain Language Statement, Consent Form, and Question Guide to ensure that they were appropriate and would be understood by study participants. After data was thematically analysed and an outline of this paper was written, they provided feedback. They then provided comments on a first draft of the paper. They were also involved in obtaining the Sangha’s response to the first draft of this article by summarising the draft to the Sangha leadership, listening to their response, and relaying it to the paper's first author.^1^

## Results

Two themes identified through the analysis were ‘context’ and ‘deciding to engage’. Context encompassed features of the researchers, the district Sangha and the research setting where engagement took place. Deciding to engage encompassed the process and content of introductory meetings between researchers and the district Sangha and their outcomes, namely whether a partnership was formed and why.

### Context

#### Invited space

Local researchers from VGKK and IPH approached the district Sangha to be involved in the PLA project. They asked the secretary of the district Sangha to call a meeting, where they could invite the Sangha to be involved in the project. The scope of the project was already set prior to engaging the district Sangha. The researchers had been awarded funding to improve implementation of safe motherhood activities for the indigenous population of Chamarajanagar district.

#### CO aims and activities

Prior to the PLA project, the district Sangha’s focus had not encompassed maternal and child health. A Sangha leader described the organisation’s purpose:

“[a]*ll this time we were not focussing on health in our Sangha. We were working on fundamental rights, forest rights and struggles. We were thinking about survival*.”

Another Sangha leader affirmed,

“[T]*he Sangha role is to help the* [Soliga] *community, to facilitate and help our community because we, if we will not do, who will do it? So we are working for our community development*.”

The Soliga people live in and around the forests of Chamarajanagar district. Global and national priorities for conserving forests and natural resources mean that large parts of Chamarajanagar’s forests are legally protected under India’s wildlife protection laws. Until recently, tribal communities like the Soliga were effectively illegal aliens on their own land. In 2006, the Forest Rights Act recognised the rights of forest-dwelling people over their traditional lands for the first time, correcting decades of land alienation among tribal people. When the Act was passed, the district Sangha studied it, developed an understanding of what its provisions meant for the Soliga people, and then started fighting for those rights. They sought to get recognition of land ownership for each family in the 148 villages that they represent.

The health of the Soliga people has largely been the purview of the government and VGKK, an NGO founded in 1980 by Dr Hanumappa Sudarshan. Dr Sudarshan not only set up a hospital for the Soliga people that was well recognised and prestigious for many years but also sought to address the social determinants of their health problems. Over time, the remit of VGKK gradually shifted away from the provision of healthcare and services to address issues the Soliga people face in relation to education, housing and sustainable livelihoods. This shift has created a need and space for other organisations to focus on the healthcare and system needs of the Soliga people.

#### Foundations for shared decision-making

A pre-existing relationship of trust, CO empowerment and CO research capacity comprised foundations for sharing power in decision-making between the researchers and the district Sangha. Prior to initiating engagement on the PLA project, the principal investigator and a co-investigator had worked as doctors in the Soliga community for many years and were known to the Sangha. One was even working for VGKK, which helped set up the Sangha organisation and continues to have a very positive relationship with it. Sangha leaders expressed their trust in the researchers, with one affirming “*they have saved the lives of our people*”.

The district Sangha is a mature, well-organised CO, given its significant experience fighting for land rights and performing community development work over many years. Sangha leaders regularly work and meet with senior government officials. This means that,

“[w]*e don’t have a fear about the VGKK or this people* [the researchers]*, our community people… Because I think now communities are empowered, so our community leaders are empowered so they can manage it. They know now they are I think they work ten to fifteen years and they have knowledge, they can manage it, they can manage the community issue… so whatever knowledge you have I’ll consider it and whatever knowledge we have or community people have we can, we will share it. You can listen*.” (Sangha leader)

A minority of district Sangha leaders also had training in research and other research-relevant skills (e.g. systematic documentation). Two had completed PhDs. A researcher interviewee remarked that having conversations about research design and methods was thus "easy" with the district Sangha leaders.

### Deciding to engage

#### Initial framing of PLA project by researchers

At the first meetings between the VGKK and IPH researchers and district Sangha, a case was made for why the PLA project focused on maternal and child health. According to Sangha leader interviewees, the researchers explained that poor maternal and child health is an important problem for the Soliga community, resulting in anaemia, underweight babies, stunting, miscarriage, and maternal and child deaths. These outcomes reflected (amongst other things) poor nutrition, poor access to health services, and babies being born to young girls and adolescents as a result of child marriage practices within the Soliga. The researchers said the PLA project would help improve maternal and child health service delivery and access for Soliga women and children. However, Sangha leaders noted that maternal and child health was described as an entry point; the PLA project could gather information about other problems in the Soliga community too. The project did not have to focus on maternal and child health alone. Numerous other issues facing the Soliga community (e.g. alcoholism, drug addiction, gangrene, cardiac problems) were also discussed during the first meetings.

#### Deliberative discussions

During the initial meetings of the PLA project, Sangha processes were followed, which meant that deliberative discussions took place. Features of these deliberations were asking questions about the PLA project and receiving answers; discussing the pros and cons of the PLA project; having an equal opportunity to speak; giving opinions and views; dissenting; making requests; and emphasising the value of Sangha leaders’ knowledge.

In the directly observed meeting of the THETA project, features of discussion similarly included asking questions and receiving answers, giving opinions, making requests, and reaching consensus. That meeting aimed to share the results of a new health survey with Sangha leaders and to get their ideas for what interventions might address some of the problems identified by the survey. There was, however, little debate of the pros and cons of proposed interventions, possibly due to role-based hierarchies of knowledge that assume the researchers know best about health matters. (We return to this later in the paper.) Much of the discussion was between the researcher leading the meeting and specific Sangha leaders. The researcher addressed questions to certain Sangha leaders more than others. Both main decisions reached at the meeting were largely proposed by the researcher leading the meeting and agreed to by the Sangha leaders.

##### Key requests

Sangha leaders made requests and asked specific questions in relation to the PLA project during initial meetings. In relation to the district Sangha’s role in the PLA project, one Sangha leader reported that the district Sangha demanded they lead data collection and analysis and that the researchers agreed:

“*Our Sangha leaders told clearly that if you give the responsibility for our Sangha we will take active part, otherwise we will not participate in this project*.”

In contrast, another Sangha leader said that the researchers defined the Sangha’s role:

“*You researchers, give us the responsibility of doing this project…We both will plan how to go about. We will give only the inputs. You have to go to the field through community. This they made it clear. We conducted that program according to that*.”

Another request was for the project’s scope to be broader than maternal and child health and for data collection to be used to identify other problems in the Soliga community as well because

“*When we are working in the community, we can’t focus only small health issue and work on that issue. When we go to the community, we have larger issues… We told* [the researchers] *… we will focus seventy-five percent for health issue. This is collection data and data thing but we’ll focus on twenty-five percent for other issues also, the same data collector will be used for other data collection*.” (Sangha leader)

The Sangha brought in certain questions and priorities to the data collection, effectively ‘piggybacking’ off the PLA project. For example, they wanted to know whether individual households had gotten their rights under the Forest Rights Act and whether they were receiving benefits from certain nutritional schemes run by the government. This request was also agreed to by the researchers.

In terms of the budget, Sangha leaders asked to see it very early on and were given full access. They further wanted to know who the PLA project funding came from, who held that money, what the different budget line items meant, and how much money they would receive to pay field investigators and their supervisors. Their queries were answered by the researchers. Questions about who controlled the budget reflected prior experience working with NGOs. Rather than following the typical NGO model, where the district Sangha spend money and then claim reimbursement, researchers suggested a memorandum of understanding (MOU) be created between the district Sangha and VGKK. The Sangha agreed. As part of the MOU, the Sangha got a first instalment of funds to start things off and then a second and third instalment based on certain deliverables being met.

At initial meetings, Sangha leaders also raised the following questions about the nature of the partnership:

- What do you mean by partnership? Who are you coming in as?
- Who makes the decisions; will we be given autonomy or not?

These questions reflected their past experience working with NGOs. Since the principal investigator worked as a doctor with VGKK, Sangha leaders wanted to know if she was representing the NGO or coming in as a researcher. If she was representing the NGO, they wanted to know whether partnership meant she would tell them what to do. A researcher reflected that this was likely

“*because I think in the past that’s how their relationship was, that the NGO brought programs and gave clear instructions, and the organisation will be the implementer. So they always implemented under the NGO, so the NGO was always like asking for reports for every money, every rupee spent and making decisions and if they decided to go left then, no the NGO would say that doesn’t make sense just go right. And so they were very wary of all these things*.”

Finally, the Sangha leaders and field investigators asked that the data collected as part of the project be acted upon. The data would speak to what problems exist in the Soliga community with respect to maternal and child health. Solutions or interventions to address one or more of those problems then needed to be designed and implemented. This also meant that the Sangha leaders and field investigators requested the project run for longer than the 1 year for which it had been funded:

“*In six months or three months, we can only collect data or information but we cannot find solution for the problems of the people in difficulty. So if the project continues for five years, it helps poor people*.” (Field investigator)

In response, the researchers said or made apparent promises that they would take up other projects to help the community after the PLA project finished.

#### Consensus-based agreement

The partnership agreement between the researchers and district Sangha had four main features – dual purpose data collection, Sangha ownership of any non-health data collected during the project, Sangha leadership of data collection and analysis, and a MOU giving the Sangha control over their portion of the budget. The first part of household interviews collected general information about every village. The Sangha owned the data collected on those topics and did not share that with the researchers.

#### Reasons CO agreed to partner

Sangha leaders and field investigators reported seven main reasons why they agreed to be part of the PLA project (Table 1). Many of the Sangha’s reasons for agreeing align with their concept of a good partner (Table 2). Interviewees also indicated their trust in the researchers to do good for the Soliga community prompted them to agree to undertake the project. That trust was linked to the researchers' many years of work at VGKK Hospital, providing healthcare to the Soliga community. Sangha leaders’ comments further indicated an assumption that the researchers, as doctors, know more about health matters than they do, which may speak to a role-based power dynamic and hierarchy of knowledge. While Sangha leaders are confident in their knowledge of forest rights, some or many are perhaps less confident in their health knowledge.

**Table 1:** Reasons the district Sangha agreed to be partners on the PLA project

Reason	Example given by Sangha leader
Addresses a community/organisational need or priority	“*When we visited our villages and podus* [hamlets]*, we found out that maternal and child health was a big health issue, most of them were anaemic and many of the babies and also some mothers would die during the delivery. We had given the information many times about this to the concerned health department, but we did not get a proper response from them, so we used to think, if anybody like this comes for help we should take it and serve the community and improve the situation*.”
Benefits the Soliga community: will improve health and help save lives	“*People in other communities live long say for 100 years also, but here if they die at 35 years only, there must be a problem in the health of our community. So to improve the health of the community, we have to take the help of the researchers*.”
Involves the community in its conduct (as partners and field investigators)	“*This is the first program like this in the district level, where everybody are included to work in the project, the same. Even VGKK is doing in its own way and government is also doing, but here we people and the organization can do the survey and go to the root of the cause for the problems of the people, so we liked this concept*.”
Aligns with the Sangha’s mission	“*So like that we, health is a very major issue for us so people are dying or people are facing health problem, they are not getting health facilities, health services, so that’s a job, the responsibility of us, all Sangha, to provide the facilities, to help the people*.”
Trust in the researchers’ expertise on health matters	“*We know* [the researchers] *from many days. We worked with them earlier also and we have full confidence in them, that they will do a good work, whatever they do. Since we had that belief, we were very happy when we heard about the project. Almost everybody know* [name] *doctor, and he has done a very good job in medical field in Karnataka, so all of us with a good heart agreed and took the decision to help the project*.”
Generates data that can be used to attract government help for the Soliga community	“*If we have the data with us we can discuss with government at any time… Their plan was to identify mother and child health problems and reach it to government and correct it. We are very much satisfied with their plan*.”
Creates a positive impression of the Sangha in the Soliga community	“*Others might think, these people are taking care of the health of the community. They take care of the health of the women in the community as their sisters*.”

**Table 2:** Features of a good research partner

Feature	Description by Sangha leader or field investigator
Understands the community, is sensitive to their problems and is service-minded, aiming to improve the community’s health through its projects and assist in other ways when it becomes aware of other problems facing the community	“*They should be ready to serve the* [Soliga] *people at any situation… They should have the mind set to develop or improve the condition of our people. They might come to know about our health condition but along with that they should understand our other problems also and share that with government or NGOs and they should try to solve the problem*.” (Field investigator)
Embedded in the community	“*They should be one among us*.” (Sangha leader)
Works with the community and community organisations in projects and helps them learn new things and develop new capabilities	“*We may not have some techniques or ideas. They should teach us those things. When will you help us grow? Other citizens think tribes will be inside the forest and they do not know anything. So you take tribes into your projects and help them to grow… You choose people among us who can mingle and work with you. Do the work with community…Involve the people from tribal group, then you will be successful*.’ (Field investigator)

## Discussion

We have presented data that speaks to the district Sangha’s experience of deciding to engage with researchers on the PLA project. We now critically reflect on that data from our positions as ethics and health systems researchers.

### Quality of the Sangha’s participation

Deliberative processes have been proposed as a way to deepen participation in democratic societies. Theories from political philosophy delineate ideal processes for deliberative decision-making [17, 18]. All participants have equal rights to initiate speech acts, to question other participants and to respond to their questions, and to open debate [19, 20]. They debate the pros and cons of various proposals until consensus is reached [18]. Similarly, equal rights of participation, asking question and obtaining answers, debating pros and cons, dissenting, and consensus were features of the discussions in initial meetings between the district Sangha and researchers, reflecting a robust deliberative process.

Ensuring equal rights of participation demands that decision-making processes be structured to mitigate inequalities in influence and power [21, 22]. This is to avoid tokenism, that is presence without voice and voice without influence. In the PLA project’s initial meetings, the deliberations may not have been sufficiently structured to mitigate the impact of researchers’ position as doctors with expert knowledge on health matters. The researchers did emphasise the value of Sangha leaders’ knowledge but perhaps more was necessary to mitigate the gap for some Sangha leaders, given certain interviewees’ comments and what was observed. For example, it may be important to directly address questions to the range of participants over the course of meetings rather than select individuals who the researchers know well. This could have helped ensure that more Sangha leaders had a say.

The data about decision-making dynamics obtained in the observed THETA project meeting were perhaps slightly different to the dynamics of the PLA project’s initial meetings described by interviewees. Both involved the Sangha making requests of the researchers and the Sangha agreeing to ideas proposed by the researchers (i.e. the MOU). In the PLA project, the final intervention selected was initially proposed by a Sangha leader. However, in the THETA project meeting, the researchers proposed the agreed-upon interventions. To some degree, this may reflect the role-based hierarchies that were previously discussed. It is also possible that the dynamics reflect the familiarity and trust built from the previous partnership experience. It is interesting to note that the quality of deliberation may have diminished slightly in terms of debating the pros and cons of interventions due to increased trust and familiarity.

A distinction is made in the literature between being able to raise one’s voice and being listened to in decision-making processes [23]. Higher quality participation means influencing the decision that is reached. In the PLA project’s initial meetings, the requests of the Sangha leaders were clearly reflected in the agreement reached with the researchers. The quality of individuals’ participation has been further connected to when individuals begin to participate and their level of participation [24, 25]. In the PLA project, the Sangha were included as decision-makers but they did not become involved in the PLA project until after funding was obtained. They were thus excluded from the grant-writing stage where the research project’s focus on improving implementation of safe motherhood activities was set. Earlier entry in the process would have enhanced their participation.

### Lessons from the Sangha experience

Despite having some areas for improvement, the Sangha experience offers other COs lessons that can help them when deciding to engage with researchers in terms of what features to look for in research partners, what features to look for in proposed research projects, what requests to make of prospective research partners, and what sorts of outcomes or partnership agreements to accept. Table 3 summarises key lessons in the first three areas.

**Table 3:** Key matters for community organisations (COs) to consider or ask when deciding to engage with researchers

**Partner features**	Sensitive to community problems
	Service minded; aims to benefit community
	Helps community grow (e.g. empowers, builds capacity)
	Embedded in community
**Project features**	Addresses a community/organisational need or priority
	Benefits the community (e.g. improving health, empowerment)
	Aligns with CO mission
	Involves CO and community in its conduct
**Key requests**	Project scope includes community needs and priorities
	Preferred level of CO participation (e.g. leadership of data collection and analysis)
	Budget transparency and control (of CO portion at a minimum)
	Project data used after collected and analysed (e.g. for advocacy, to design interventions that are then implemented)
	Data access and ownership
	Long-term projects and sustained partnership
	Partnership characterised by joint decision-making
	Capacity development

With respect to outcomes, most of the requests made by the Sangha were agreed to by the researchers. This included larger requests that affected the project’s agenda and the nature of the partnership as well as smaller requests (e.g. for training). When considering if a partnership agreement is acceptable, COs should reflect on whether those requests that are especially important to their organisation have been met and, if not, whether compromise decisions are satisfactory and worthy of agreement.

Ultimately, the case study suggests that meaningful engagement and shared decision-making can occur when research partners and research projects have the features described in Table 3, key requests are deliberatively discussed and consensus is reached on them. We further note that certain contextual features of the district Sangha experience are reflected in these lessons and should be born in mind. First, researchers initiated the engagement in the PLA project, rather than the CO inviting them to collaborate on a project of its choosing. This is consistent with much current practice, where researcher-initiated collaborations are more common than CO-initiated partnerships [12]. The PLA project topic and funding were already organised prior to engagement, spurring Sangha leaders to ask questions about its scope and budget. While we do not suggest such arrangements are ideal, at present, engagement usually occurs after research funding is acquired. We think the lessons from this case study would thus apply to much current engagement practice in health research. However, additional/alternative key requests and questions may apply in CO-initiated partnerships and this requires further exploration.

Second, in the PLA project, relationships of trust existed, the CO was empowered and comfortable discussing research matters, and had many of the skills needed to lead data collection and analysis. Where relationships of trust have not yet been built or COs are less empowered and skilled, alternative/additional questions and requests may be relevant that the paper has not captured, and acceptable outcomes may look slightly different to what has been presented. Such matters also require further examination.

### Comparison of study findings to existing guidance

These findings are consistent with the limited existing guidance on forming research partnerships. Such guidance primarily speaks to two contexts, namely (1) academic–community partnerships in community-based participatory action research and 2) high-income country and low- and middle-income country partnerships in global health research.

To assist low- and middle-income country institutions to enter more equitable partnerships, the Council on Health Research for Development (COHRED) has developed *Guidance for Fairer Contract Negotiation in Collaborative Research Partnerships*. This guidance document identifies five key topics to address in negotiations in order to generate more equitable partnerships: intellectual property, capacity-building and technology transfer, ownership of data and samples, compensation for indirect costs, and contracts [26]. Similarly, the Sangha experience identified capacity-building and data ownership as key requests (amongst others) to make and the matter of CO compensation was a key topic raised with researchers. In the Sangha experience, concern was for budget transparency and control and ensuring the Sangha portion of the budget covered the project costs it would incur. A 10% indirect cost was included in the budget. Contracts were addressed via the development of a MOU between VGKK and the district Sangha. However, the COHRED guidance document describes many other forms of contract [26]. It may be useful for other COs to consider this broader spectrum of contracts and determine which best apply to meet their needs.

The South Carolina Clinical and Translational Research Center for Community Health Partnerships has developed a guidance document and toolkit for assessing community–academic partnership readiness [27]. It calls for prospective partners to first assess their fit as partners. Doing so entails having open discussions about what their values and missions are and whether they align; trust and any past negative history between the partners, their organisations and the community; what each partners’ people, processes and structures look like; whether the timing is right for both partners to collaborate; how the partnership will benefit both partners; and their commitment to the project (time and resources) [27]. Similarly, Sangha leaders and field investigators identified aligning with the CO mission, benefiting the community, and helping the community and CO grow as key project features. Trust was identified as a key partner feature and the Sangha’s past experiences partnering with NGOs were reflected in the questions they raised during initial meetings with researchers. Nonetheless, the key requests and questions identified by this case study research could be supplemented by the discussion of the following topics from the toolkit: values and missions, past history, mutual benefit, and partner processes and structures.

### Study limitations

The case study had several limitations. Ideally, more women, more Sangha leaders from Chamarajanagar and Gundlupet sub-districts, and more non-Sangha field investigators should have been interviewed. The latter were sought to offer more impartial perspectives on whether the CO was a good representative of the Soliga community. Efforts were made to interview individuals in these categories but finding available interviewees proved difficult.

Given that the case study was carried out more than 4 years after the PLA project started, several interviewees had poor recall of what happened during the project’s initial meetings. Where this happened, it was noted in the field notes for the particular interview and it was then considered when coding the data. Parts of interviews were excluded where an interviewee did not remember an event or were coded under the category ‘general Sangha processes’, where an interviewee described how things generally happened in Sangha meetings rather than what had happened in the initial PLA project meetings. The retrospective nature of the case study also meant that initial meetings between the researchers and Sangha leaders were not directly observed. To supplement interview data, a 2019 THETA project meeting between Sangha leaders and the VGKK and IPH researchers was observed. It is possible that BP and NSN’s presence at that meeting affected the observed dynamics between the researchers and the Sangha.

There was potential for Kannada-speaking interviewees’ comments to be misinterpreted during analysis. After interviews done in Kannada were translated to English, the lead author of the paper, at times, still found it difficult to understand the points interviewees were making. Where questions of interpretation arose, the lead author relied on NSN to provide clarification. NSN works for IPH but was not part of the PLA project. To ensure that the quotes used in the paper reflected their intended meanings, the PLA researchers and Sangha leaders were also given early versions of the paper to read and provide comments.

## Conclusions

Achieving deeper forms of community engagement in health research demands decision-making be shared with community partners. However, how can community partners assess if meaningful engagement and shared decision-making will be possible when approached by prospective research partners? Limited guidance exists on this matter. As such, this paper sought to identify key considerations for COs to take into account when deciding to engage with researchers (Table 3). We hope that this guidance will help empower COs to select good research partners and promote more equitable partnerships between them and academic researchers.

## Data Availability

The datasets used and/or analysed during the current study are available from the corresponding author on reasonable request.

## Abbreviations

CO: community organisations
MOU: memorandum of understanding
NGO: non-governmental organisation

## Footnotes

1 The Sangha leaders appreciated the details but did not have specific comments on the paper itself.
